# Accuracy of fit for cobaltchromium bar over two implants fabricated with different manufacturing techniques: an in-vitro study

**DOI:** 10.1186/s12903-023-03700-w

**Published:** 2023-11-29

**Authors:** Hossam I. Nassar, Ayman Fateen

**Affiliations:** Faculty of Oral and Dental Medicine, Future University in Egypt, Cairo, Egypt

**Keywords:** CobaltChromium, Implant, Bar, Milled bar, 3D printing, Internal fit

## Abstract

**Objective:**

The purpose of the invitro research was to compare the fit of Cobalt Chromium customized bar fabricated with different manufacturing processes cast metal bar, milled bar and 3D printed bar using scanning electron microscope.

**Materials and methods:**

Clear epoxy resin molds were prepared. In each mold two parallel implants with a 14 mm distance from each other were embedded. Thirty Co-Cr custom bars were constructed and were divided equally into three groups: Group (I) (Co-Cr conv), group (II) milled bar (Co-Cr milled), and group (III) printed bar (Co-Cr print). The marginal fit at implant-abutment interface was scanned using scanning electron microscope (SEM).

**Results:**

There was a significant difference between the three studied groups regarding marginal misfit the between implant and fabricated bars with p-value < 0.001. The highest value of micro-gap distance was found in Co-Cr conventional group (7.95 ± 2.21 μm) followed by Co-Cr 3D printed group (4.98 ± 1.73) and the lower value were found in Co-Cr milled (3.22 ± 0.75).

**Conclusion:**

The marginal fit of milled, 3D printed and conventional cast for Co-Cr alloy were within the clinically acceptable range of misfit. CAD/CAM milled Co-Cr bar revealed a superior internal fit at the implant-abutment interface. This was followed by selective laser melting (SLM) 3D printed bar and the least fit was shown for customized bar with the conventional lost wax technique.

## Introduction

One of the most important factors for the success of treatment planning in screw retained implant prosthesis is directly related to the passivity of the restoration. The passive fit of restoration after the final tightening of all screws can be achieved when the opposing surfaces of the implant and the prosthetic component are in maximal three-dimensional resemblance and contact without strain in any of the components [1, 2].

The fit and the micro gap between the implant and customized bars can lead to both mechanical and biological complications. The colonization of microorganisms in the inner part of the implant can eventually cause inflammation of peri-implant tissues and bone strain with consequent pain. This situation may lead to marginal bone loss around the dental implant and eventually can lead to failure of the whole osseointegration [3, 4].

The increase in the number of implants placed, their angulation, and the distance between them can increase the risk of misfit of implant components. Besides, the conventional workflow for final restoration starting from the impression procedure involving impression analogues, impression materials, and stone casts can even potentially grow the problem [5].

Implant abutments for different prosthetic restorations are either prefabricated (stock) which is commercially available or customized [6]. The customization of bars and abutments can be produced by conventional cast procedure such as UCLA-type abutment. Castable UCLA abutments are usually combined with a prefabricated metallic base of cobalt chromium that fits exactly in the internal connection of the implant to prevent distortion and misfit that can arise in the cast procedure [7].

In an attempt to control internal misfit between the implant and custom-made abutments and bars from a fully digital workflow, the use of hybrid abutments has evolved. This concept is known as Ti-Base abutment which consists of an industrially prefabricated abutment system that can accept custom milled monolithic or bi-layered superstructures and different metallic bar design restorations [8, 9].

The abutment–implant interface is considered one of the areas where occlusal force is concentrated and transferred to the implant. Therefore, long-term firmness is crucial for reducing clinical complications of dental implant. Lack of precision fit at the implant-abutment interface leads to prosthetic complications affecting the meso-structure or the superstructure. The complications include increase of occlusal overload, loss of passivity, micro-Pump effect, peri-implantitis, frequent screw loosening, abutment screw fractures, wear and deformation of the implant index, wear of the abutment connection and loss of implant osseointegration [10–13]. Chrome-Cobalt alloy (Cr-Co) was largely employed in several aspects in dentistry due to its high strength, low cost, corrosion resistance when compared to other alloys. Unfortunately, scientific research concerning the use of Co-Cr alloy for implant restorations is inadequate [14].

Various techniques were generally discussed to evaluate implant-abutment fit in literature. Clinically, it can be evaluated by visual inspection, radiographs, and alternative finger pressure for tactile sensation of the joint. Sheffield test and screw resistance test were claimed to be highly precise for any lack of passivity and presence of implant abutment misfit. Microscopic methods of evaluation have also been employed including the use of a light microscope and scanning electron microscope (SEM) to measure the implant abutment micro gap fit. The micro gap fit can also be assessed through finite element modelling, strain measurement, photoelastic stress analysis, or by digital scan of surface superimposition for the final framework and the master cast [15, 16].

Today, computer-aided designing and computer-aided manufacturing (CAD/CAM) enabled the wide use of custom-made abutments and bars with the following advantages: reduction of fabrication time, precise level of customization and wide selection of different manufacturing materials. CAD/CAM technology has been recommended as a development over conventional cast manufacturing because it allows the high accuracy machining of prefabricated blocks of different materials including composite and acrylic resins, ceramics, and different metal alloys as titanium and cobalt chromium [17].

Research work and industrial procedures concerns determining whether the additive manufacturing technologies can substitute or introduce a new manufacturing system [18, 19]. Those additive manufacturing technologies fabricate customized subperiosteal, endosseous titanium implants [20–24], meshes for different bone grafting procedures, (25–26) and Co-Cr and Titanium frameworks for either fixed or removable implant-supported prostheses [27–29].

The purpose of the present invitro study was to compare the marginal fit of Co-Cr customized bar fabricated with different manufacturing techniques. The null hypothesis was that there is a difference in the marginal fit of Co-Cr customized bars fabricated with digital and conventional processes.

## Materials and methods

### Specimen preparation

Thirty clear epoxy resin molds (Swiss Chem: construction chemicals, Egypt) were prepared and used in the present study in the following dimensions: 8 mm in height, 18 mm in width, and 8 mm in thickness. In each mold two bone level dental implants (Neobiotic, IS-II active, South Korea) with internal morse connection in its upper part and hexagon shape in its lower part as anti-rotation feature. The implants were 4 mm in diameter and 10 mm in length and they were embedded leaving 3 mm of implant surface exposed. The implants were placed parallel to each other by the aid of dental surveyor (Ney Surveyor, Dentsply, USA) with a 14 mm distance from each other. Two scan abutments (Neobiotic, IS scan body, D4, SCRP, South Korea) were attached to the implants and scanned using a desktop scanner (D850, 3Shape, Copenhagen, Denmark). The STL files were imported, and a bar over the two implants was designed using 3Shape software program (3Shape dental designer, 3Shape A/S, Copenhagen, Denmark). A total of thirty bars were manufactured by different manufacturing techniques as follows.

Twenty Co-Cr bars were constructed by milling process utilizing the computer milling machine (CAM) (Ceramill, Amann Girrbach, Austria) where the cutting tools remove the excess material gradually and shape the bar according to the planned design (CAD). The twenty bars were sub divided equally into: Group (I) (Co-Cr conv) were polymethyl methacrylate (PMMA) (Yourcera Biotechnology Co.,Ltd, China) milled bars acquired from the previous scanned and designed data and later the metallic bars were obtained by conventional lost wax technique using Co-Cr alloy (Scheftner Dental Alloy, GmbH, Germany). In Group (II) (Co-Cr milled) were Co-Cr milled bars directly milled according to the previous design.

In Group (III) (Co-Cr print) ten bars were obtained by selective laser melting (SLM) 3D printed process. The 3D printed bars (SLM) were fabricated using the laser beam (HBD 100D, Guangdong Hanbang 3D Tech Co. Ltd, China), in which the raw powdered material of Co-Cr was placed in a tray (Scheftner Dental Alloy, GmbH, Germany). Later, laser beam was spotted over the tray to rise the temperature of the powder and bind the particles together layer by layer to the required shape. Laser power was set to 200 W, the speed of laser scan to 1200 mm/s, the hatch distance to 0.03 mm, and finally layer thickness of 30 μm was applied. Following the SLM processes, the specimens were exposed to heat treatment in a furnace with high-purity argon. The heat treatment in the furnace went from room temperature to 1000 °C in one hour and kept at that temperature for an additional one hour. Then the specimens were slowly cooled to room temperature in the furnace.

**Fig. 1:**
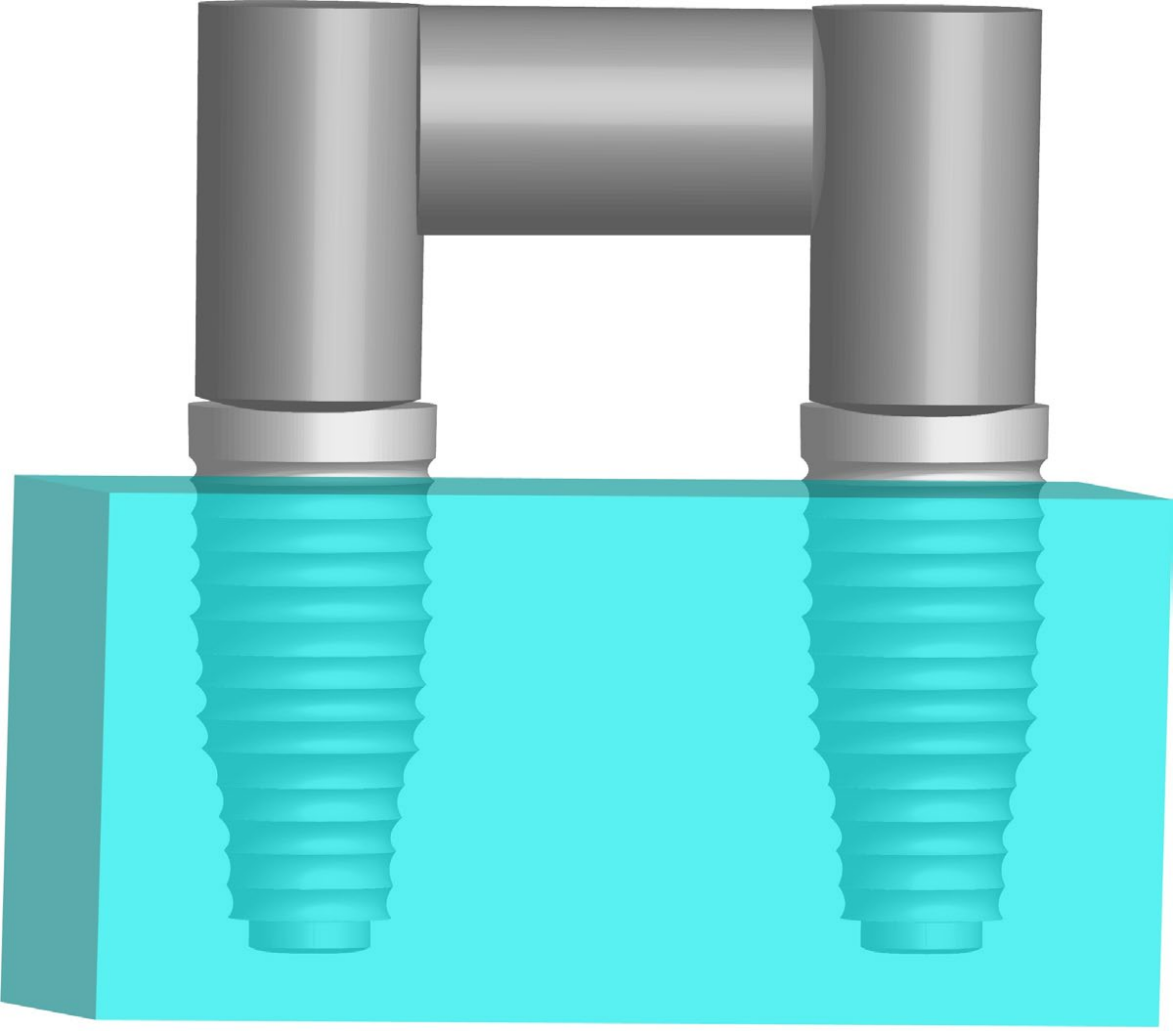
Two implants inserted in the resin block with the manufactured bar fitted into the implants engaging their internal connection

**Fig. 2:**
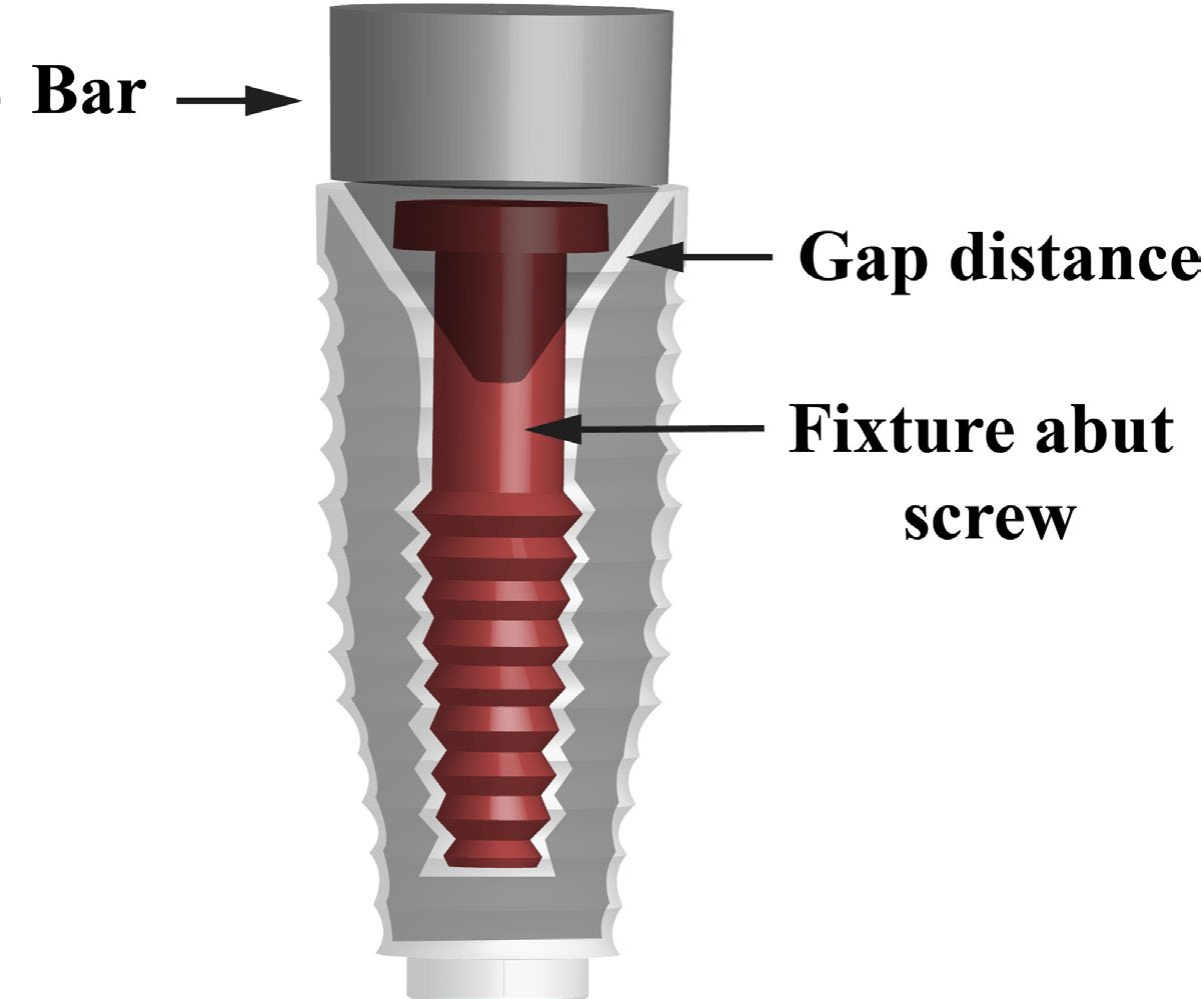
Vertical cross section of implant showing internal gap between custom bar and implant after tightening of abutment screw

The different manufactured bars were then fitted into the implants engaging their internal connection and the abutment screw was tightened using a dynamic torque wrench of 30 N following the manufacturer’s specifications (Fig. 1). All implants were subjected to vertical sectioning using water jet-powered sectioning equipment (Isomet, Buehler, Germany), followed by copious rinsing with distilled water and ethyl alcohol to remove any clogged debris that would affect accurate visualization of the implant–abutment interface. All samples were then cleaned in ultrasonic bath for 10 min (Beijing Ultrasonic Co., Beijing, China). Finally, all the test specimens were washed thoroughly with ethyl alcohol and dried (Fig. 2).

### Fit evaluation

**Fig. 3:**
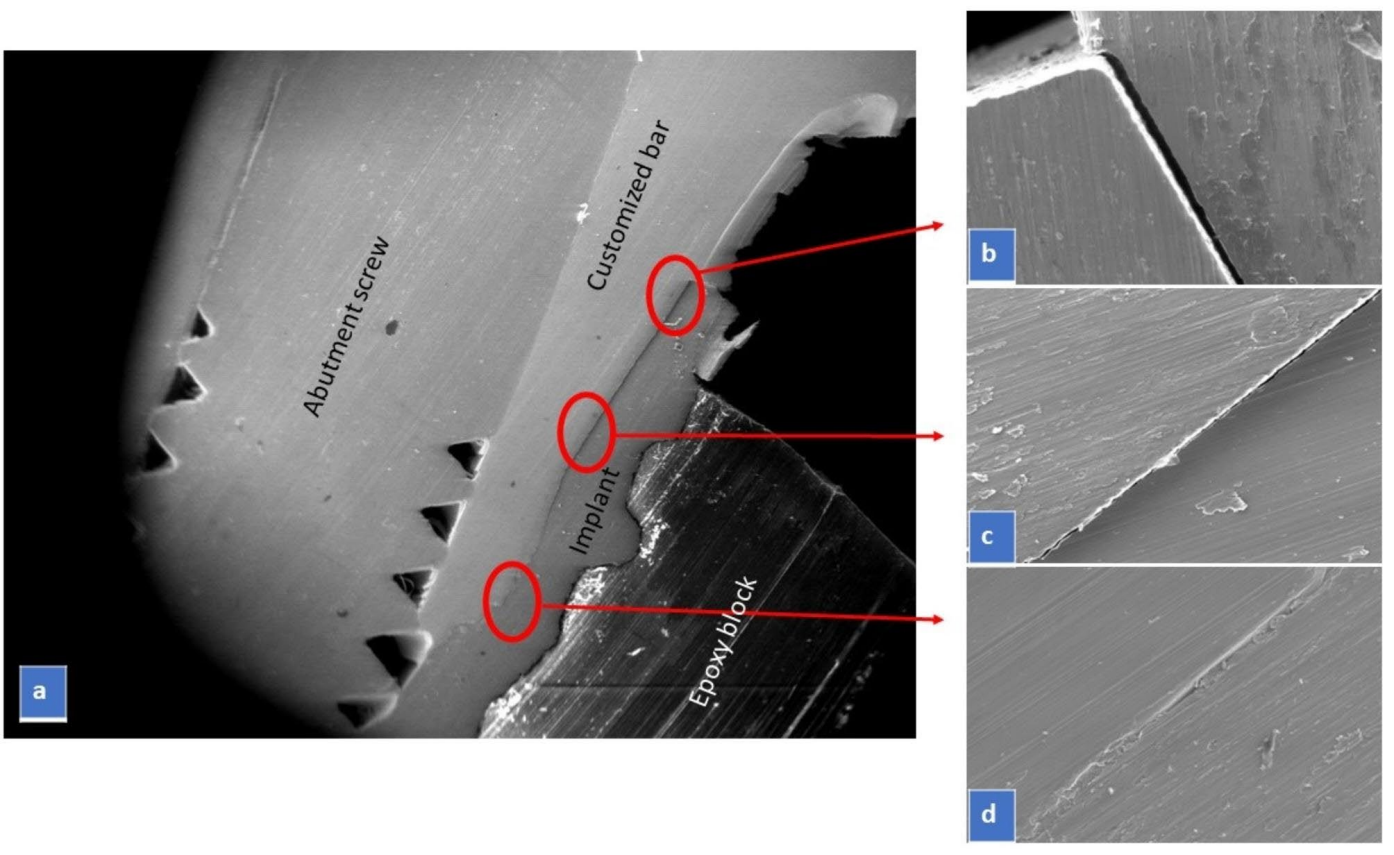
(**a**) Vertical cross section of customized bar-implant interface and the three (top, middle, and bottom) on each side measuring areas as illustrated by the red boxes with SEM at 52× magnification (**b**), (**c**), and (**d**) are sample electronic photomicrographs at 800× magnification for measuring gap distance

The marginal fit at implant-abutment interface of each prepared sample was scanned by using high resolution scanning electron microscope (SEM), the analysis was carried out on a FEI Quanta FEG 250 (Thermo Fisher Scientific, Eindhoven, The Netherlands) instrument. In each sample, 3 measuring points on each side (top, middle, and bottom) with total 6 points at the implant– abutment interface, were selected for measurement of the vertical marginal fit between the abutment margin and the internal conical connection surface angle of the implant platform. The selected working parameters for the samples were at 800× magnification and the photomicrographs were obtained in separate images to aid in accurate measurement of the fit. The interface fit was then measured on the scanning electron microscopic images obtained for each test sample using an image measuring pixel counting software (Image J, National Institutes for Health). The fit was then measured on the SEM images with the linear measuring scale of the software (Fig. 3).

### Statistical analysis

Data were collected, revised, coded, and entered onto the Statistical Package for Social Science (IBM SPSS) version 23. Data were described as mean and standard deviations. Using one-way analysis of variance (ANOVA) test, the comparison between groups were made followed by post hoc test using least significance difference (LSD). The confidence interval was set to 95% and the margin of error accepted was set to 5%. The p-value was considered significant at the level of < 0.05.

## Results

There was a significant difference between each of the three studied groups (I vs. II, II vs. III & I vs. III) regarding marginal misfit the between implant and fabricated bars with p-value < 0.001. The highest value of micro-gap distance was found in Co-Cr conventional group (7.95 ± 2.21 μm) followed by Co-Cr 3D printed group (4.98 ± 1.73) and the lower value were found in Co-Cr milled (3.22 ± 0.75) (Table 1).

**Table 1:** Number of readings and mean gap distance from SEM taken for the whole sample used in the experiment

Grouping	Manufacturing Technique	Number of implants	Total number of readings	Mean ± SD (µm)	P-value
Group (I)	Co-Cr conv	20	20 × 6 = 120	7.95 ± 2.21	< 0.001
Group (II)	Co-Cr milled	20	20 × 6 = 120	3.22 ± 0.75	
Group (III)	Co-Cr print	20	20 × 6 = 120	4.98 ± 1.73	

## Discussion

The rationale behind comparing different manufacturing techniques in bar cases used in this study is built on the increasing demand for customization of abutments for different clinical conditions. Generally, custom abutments allow the clinician to develop the prosthesis emergence profile with customization of the cervical margins comparable to the anatomy of a natural tooth and to compensate for improper implant angulation. Furthermore, custom made abutments can be fabricated out from different materials including metal alloys as Titanium and Cobalt Chromium, ceramics, and resin materials [30, 31].

Taking into consideration that the abutment-implant interface plays a major role on the success and long-term stability of implant-supported rehabilitations, it becomes paramount to inspect the influence of different manufacturing methods on the internal fit and accuracy at the implant-abutment interface.

Technically, gaps and misfits between the implant and abutment are unavoidable. It is impossible for such components to accurately match with zero gap because of the precision limitation during the manufacturing procedures and some amount of micro leakage will be inevitable regardless of the type of connection. The amount of these micro gaps and their clinical significance have been getting remarkable attention [4, 7, 32, 33]. Accordingly, different proposed methods have been employed to measure those micro gaps including the use of direct visualization under optical microscope [34], or traveling microscope [35] microtomography [36], and the analyzing method under scanning electron microscopy (SEM) [37], for either sectioned or embedded specimens and the use of a silicone replica. Although these techniques are well recognized, nevertheless, it is impossible to be used in vivo and several authors agreed that these measurements could involve unavoidable human errors, along with the non-standardized evaluation areas [38, 39]. The size of the micro-gaps has been described to range from 1 to 60 μm [34, 39]. The size of micro-gap could be related to manufacturing process, implant system used, and the torque applied to fix the abutments to the implant [32, 40].

The available scientific reports concerning the use of Co-Cr alloy in either single or multiple implant restorations is scarce. Although, the precision fit of different materials as titanium and zirconia abutments has been widely studied [41, 42]. Fernandez et al. [43] compared the marginal fit of Co-Cr custommade abutments with implant having an external hexagonal connection utilizing three different manufacturing procedures: the conventional cast, printing, and milling in an invitro study. They reported the least marginal misfit of implant abutment interface in milled abutments (0.73 μm), followed by cast abutments (9.09 μm) and printed abutments (11.30 μm) with no statistically significant difference between cast and printed abutments. Gonzalo et al. [44] reported the marginal fit in both milled titanium and printed Co-Cr abutment in internal hexagon connection design for different implant systems at implantabutment interface. The mean marginal misfit for the milled titanium abutments was reported to be (0.75 ± 1.27 μm) while in the CoCr laser printed abutments was (11.83 ± 13.21 μm).

The results obtained in the present study agreed with the null hypothesis that there is a difference exists in the marginal fit of Co-Cr customized bars fabricated using different manufacturing processes. Significant differences were observed in the studied groups. Several studies reported that the micro gap of less than 10 μm should be considered acceptable [45, 46]. The marginal fit of all tested groups was within the clinically acceptable range. The marginal fit of the milled Co-Cr was 3.22 ± 0.75 μm, printed Co-Cr 3D was 4.98 ± 1.73 μm and for conventional Co-Cr was 7.95 ± 2.21 μm. Although the differences may be statistically significant but may not be clinically relevant.

The possible clarifications for the marginal misfit of the milled Co-Cr (3.22 ± 0.75 μm) could be related to factors associated in the milling process itself. These factors could be the alteration in the radius of the instruments during the milling procedure, the wear of the milling tools and the size of the milling drills [47]. Mobilio et al. [34] reported that the tolerance set for fabrication of either screw retained abutments or crowns during CAD/CAM procedure could have a major role in their final fit to implant. The study claimed that reduction in tolerance values by 10-µm could increase the attrition and, subsequently, the vertical fit between the components.

The marginal misfit of the printed Co-Cr bar was (4.98 ± 1.73 μm). The highest marginal misfit was recorded for the conventional cast Co-Cr bar (7.95 ± 2.21 μm). Several literatures reported that laser sintering could cause some distortion and porosity that generate rough connection between bar and implant creating micro gaps and inhibiting complete passive fit [48, 49]. It was also reported that radiographic evaluations of Co-Cr dental alloys fabricated using casting, milling, and sintering procedures revealed minimal porosities on the milled and sintering specimens and gross porosities on the cast specimens [50]. This might be expected regarding the expansion of investment materials used that could cause some distortion in the implant-abutment fit [10, 22].

One of the limitations of the present study was that it’s an invitro study involving only internal implant connection. Additional points for future investigations can focus on the influence of implant connection either external or internal connections on marginal fit. Also, the role of different bar materials as titanium alloys and zirconia. The limitation of the present study was utilizing the vertical cross-section technique to measure the gap distance and the fit. As a result, the precision was assessed only at a few defined areas per each implant, and this might not totally demonstrate the complete geometry of internal fit. Cross sectioning procedure by itself could cause some damage to the specimens. Also, the effect of cyclic loading on the precision of fit was not included in the study. Furthermore, research evaluates the impact of marginal misfit on biomechanical performance in vivo and their clinical significance.

## Conclusions

Within the limitations of this study, the following conclusions may be drawn: The marginal fit of milled, 3D printed and conventional cast for Co-Cr alloy were within the clinically acceptable range of misfit. CAD/CAM milled Co-Cr bar revealed a superior internal fit at the implant-abutment interface. This was followed by selective laser melting (SLM) 3D printed bar and the least fit was shown for customized bar with the conventional lost wax technique.

## Data Availability

The datasets used and/or analyzed during the current study are available from the corresponding author on reasonable request.
